# The role of metabolic adaptation to nutrient stress in pancreatic cancer

**DOI:** 10.15698/cst2018.12.166

**Published:** 2018-11-15

**Authors:** Abhishek Derle, Maria Chiara De Santis, Luca Gozzelino, Edoardo Ratto, Miriam Martini

**Affiliations:** 1 Department of Molecular Biotechnology and Health Science, Molecular Biotechnology Center, University of Torino, Torino, Italy.; # Contributed equally to this manuscript.

**Keywords:** pancreatic cancer, nutrient stress, metabolic rewiring, autophagy, macropinocytosis

## Abstract

Pancreatic cancer is the fourth most common cause of cancer-related mortality, with a dismal prognosis that has changed little over the past few decades. Despite extensive efforts in understanding the oncogenetics of this pathology, pancreatic cancer remained largely elusive. One of the main characteristics of pancreatic cancer is the reduced level of oxygen and nutrient perfusion, caused by the new matrix formation, through the activation of stromal cells (desmoplasia). This stromal reaction leads to metabolic adaptations in surviving tumor cells in order to cope with these challenging conditions. The oncogenic signaling driven by KRAS mutation is necessary to fuel pancreatic tumors by activating key metabolic processes, including enhanced glycolysis and glutamine consumption. Here we review our current understanding of the pancreatic cancer metabolism as well as discuss recent work pointing to the importance of various metabolic strategies as well as autophagy and macropinocytosis as critical nutrient supply pathways. The elucidation of these metabolic networks may highlight new opportunities to further develop novel therapeutic strategies.

## INTRODUCTION

Pancreatic cancer is the fourth leading cause of cancer death in the developed world [1]. The most common subtype of pancreatic cancer is pancreatic ductal adenocarcinomas (PDACs) that arise from the exocrine pancreas. The lack of progress in early diagnosis and effective therapies are the main reasons why improvements in PDAC death rates have been so scarce. An early event during malignant transformation is the acquisition of activating mutations in the KRAS oncogene which occurs in >90% of PDAC patients [2]. PDACs are highly “addicted” to this oncogene for multiple parameters influencing tumor initiation, progression and maintenance as demonstrated using genetically engineered mouse (GEM) models and human PDAC cell lines [3]. Recent studies have shown that oncogenic KRAS promotes metabolic rewiring to support increased energetic and biosynthetic demand by reprogramming glucose/glutamine metabolism and increasing autophagy and macropinocytosis [4, 5].

Autophagy is an evolutionarily conserved membrane-mediated process that delivers cytoplasmic constituents to lysosomes for degradation and component recycling. This process is triggered by nutrient shortage, protein damage, and oxidative stress occurring through the inhibition of the mammalian target of rapamycin (mTOR) pathway.

In addition to autophagy, cancer cells can also absorb extracellular components through an endocytic process of macropinocytosis. Macropinocytosis is a non-specific bulk internalization of large portions of extracellular fluid [6]. Firstly, the plasma membrane protrudes through actin filaments polymerization and entraps extracellular fluid. Subsequently, it folds inwards and by fusing with the basal membrane, it forms a large endocytic vesicle, the macropinosome. Finally, these macropinosomes undergo different steps of modification to shrink and concentrate their contents and are recycled to the cell membrane or fused with the lysosome for degradation. The latter mechanism is fundamental in cell metabolism since it permits the release of digested endocytosed material, predominantly in the form of aminoacyls and lipids [7].

This review gathers the recent findings on the role of metabolic adaptation to nutrient stress that cancer cells undergo during the development of pancreatic cancer. Importantly, we will focus on the currently available and potential therapeutic strategies aimed at improving the treatment of this life-threatening disease.

## PANCREATIC CANCER METABOLISM

The metabolism of pancreatic cancer is largely adapted to support the high rate of proliferation and the synthesis of substrates necessary for tumor growth in its nutrient-deprived environment [5]. PDAC reprogramming is mainly driven by mutations in the oncogene KRAS [8–10]. In addition, other oncogenes and tumor suppressors, such as SMAD, Myc and p53, regulate the cellular metabolic state and are frequently mutated in PDAC [2, 9, 11, 12]. The metabolic rewiring and different adaptation strategies implemented by PDAC are discussed below.

### Warburg effect

The Warburg effect is one of the most established phenomenon observed in several tumors, in which cancer cells increase the rate of glycolysis even in the presence of oxygen [4], with glucose used not only as a mitochondrial source.

**Figure 1:**
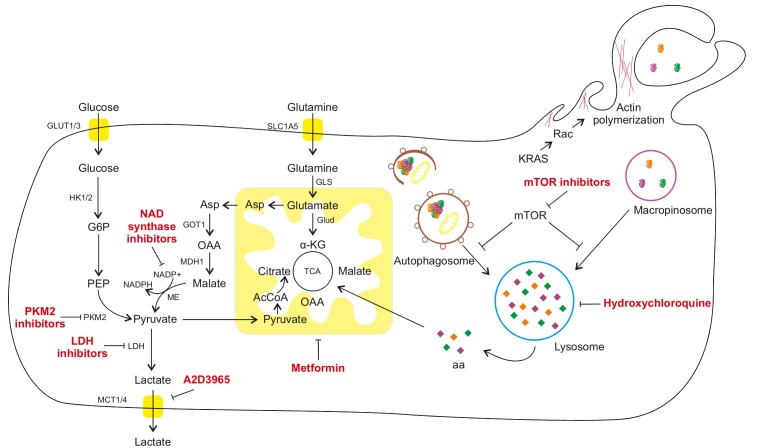
FIGURE 1: Macroautophagy and macropinocytosis as pro-survival mechanisms to sustain cancer cell metabolism in a nutrient limiting environment. Under nutrient rich conditions, cancer cells take up glucose and amino acids through membrane transporters and they can be used to support glycolysis and tricarboxylic acid (TCA), producing ATP and intermediates for anabolic processes. However, PDAC is completely surrounded by a fibrotic barrier, thus limiting the nutrients' delivery to the tumor cells. For this reason, PDAC cells develop two different lysosomal degrading mechanisms. Macroautophagy is a catabolic degradation of damaged cytoplasmic components (organelles and macromolecules), while macropinocytosis is a catabolic degradation of extracellular components previously internalized by the cell. Both mechanisms can produce amino acids and other nutrients that can fuel cell metabolism and sustain survival and proliferation. α-KG: α-ketoglutarate; AcCoA: acetyl coenzyme A; Asp: Aspartate; G6P: glucose-6-phosphate; GOT1: Glutamic-oxaloacetic transaminase 1; GLS: Glutaminase; GLUD: Glutamate Dehydrogenase; GLUT: Glucose Transporter; HK 1/2: hexokinase 1/2; LDH: lactate dehydrogenase; MCT 1/4: Monocarboxylate transporter 1/4; MDH1: Malate Dehydrogenase 1; ME: Malate dehydrogenase; mTOR: mammalian target of rapamycin; NADPH: nicotinamide adenine dinucleotide phosphate; OAA: oxaloacetate; PEP: Phosphoenolpyruvate; PKM2: Pyruvate kinase isozymes M2; SLC1A5: Solute Carrier Family 1, member 5; TCA: tricarboxylic acid.

Several oncogenic mutations affect aerobic glycolysis. In pancreatic cancer, KRAS upregulates GLUT1 (glucose transporter 1), as well as HK1 (hexokinase 1), HK2 (hexokinase 2), PFK1 (phosphofructokinase 1) and LDHA (lactate dehydrogenase A), which are key enzymes for the glycolytic processes (**Figure 1**). Nevertheless, the increased glycolytic flux is not accompanied by significant alterations in the amount of tricarboxylic acid (TCA) cycle intermediates, suggesting that glucose is primarily diverted to anabolic pathways, such as pentose phosphate pathway (PPP). The upregulation of LDHA pushes the conversion of pyruvate towards lactate, which can be secreted and taken up by more oxygenated neighbouring cells [13]. Hypoxic PDAC cells further promote the production of lactate by activating HIF-1, which negatively regulates the expression of pyruvate dehydrogenase [14]. The elevated rate of glycolysis promotes the shuttle of glycolytic intermediates towards the PPP. This pathway is composed by two distinct branches, called oxidative and non-oxidative, which contribute to fatty acid synthesis and NADPH production or nucleotide and glycolytic intermediate synthesis, respectively [15]. Moreover, p53 inhibits the glucose-6-phosphate dehydrogenase (G6PD), the first enzyme in the PPP pathway [11] and its loss in PDAC further promotes glucose consumption through the PPP pathway.

### Glutamine addiction

Glutamine is considered as an essential amino acid for highly proliferating cells since it supports the synthesis of large amounts of metabolites. In the case of PDAC, it has been recently demonstrated that its metabolism is strongly dependent on glutamine as a replenishment for TCA cycle [4, 16, 17]. In the canonical pathway, glutamine enters the mitochondria and is converted to glutamate and subsequently to α-ketoglutarate (α-KG) by the enzyme glutamate dehydrogenase 1 (GLUD1; **Figure 1**). α-KG is then metabolized in the TCA cycle and can stimulate cell growth through the mTORC1 pathway [18]. However, it has been demonstrated that oncogenic KRAS can downregulate GLUD1, favoring a non-canonical pathway for glutamine. The glutamine-derived glutamate is converted to aspartate by Glutamic oxaloacetic transaminase (GOT) 2, which is then released in the cytoplasm and sequentially metabolized by GOT1 (which is upregulated by KRAS) along with malate dehydrogenase (MDH1) and malic enzyme (ME1) to generate pyruvate and NADPH. This pathway is crucial to maintain the reducing power of PDAC cells, since it is the major source of NADPH. In fact, the oxidative branch of the PPP is strongly suppressed by KRAS, as previously described [9]. If glutamate metabolism is experimentally impaired, PDAC cells show reduced growth and increased oxidative stress [18]. Moreover, glutamine withdrawal or GOT2 inhibition may promote cell senescence through p27 pathway which is activated due to increased oxidative stress [19, 20]. Interestingly, the fact that non-transformed cells are not affected by glutamine withdrawal, further demonstrates the addiction of PDAC cells to this pathway [21].

### Hypoxic adaptation

In the last years, significant advances have been made to explain how PDAC enables metabolic reprogramming to maintain adequate intracellular nutrient levels despite limited external supply and promoting survival of cancer cells. To overcome low nutrient availability, PDAC cells overexpress several transporters to increase the efficiency of nutrient intake. The stabilization of HIF1, together with the oncogenic activity of KRAS and the loss of p53, leads to the upregulation of glucose transporters and glycolytic enzymes [4, 22]. Moreover, hypoxic cells produce and secrete more lactate, which contributes to the acidification of the tumor's microenvironment, dissolving adjacent fibers and facilitating tumor cells spread towards more oxygenated surroundings [23]. As described above, lactate can also be used by more oxygenated cells, giving rise to a symbiotic loop between hypoxic and normoxic cells [13]. Recent findings suggest that stromal cells can also support tumor growth by releasing non-essential amino acids [24]. In particular, stroma-associated pancreatic stellate cells are reported to secrete alanine, which can replace glucose as a source of carbon for the TCA cycle (**Figure 1**). Finally, some non-canonical strategies to increase nutrients availability are often upregulated in PDAC, such as macropinocytosis or autophagy, as described in the following paragraphs. The acquired dependence of PDAC on these pathways creates new vulnerabilities that can be therapeutically targeted.

## AUTOPHAGY: THE CONUNDRUM OF PANCREATIC CANCER

Autophagy is an intracellular homeostatic process which plays a crucial role in catabolic degradation of damaged cytoplasmic components by delivering them to lysosomes. Several studies indicate paradoxical role of this process in the pathogenesis of cancer, depending on tumor type and stage. On one hand, it exerts tumor suppressive functions during early stages of tumorigenesis [25, 26] by preventing the metabolic stress and chromosome instability. On the other hand, in already established tumor cells, it assists tumor growth and survival by providing an alternative source of energy and metabolic fuels [27, 28].

Recent reports exclusively points to a profound requirement of autophagy during advanced stages of PDAC progression in a cell autonomous manner. Loss of autophagy either by genetic means or by pharmacological inhibition suppresses pancreatic tumor cell growth in xenograft as well as GEM models [29]. A study conducted by Rosenfeldt, *et al.* has demonstrated that deletion of autophagy related genes (ATG), Atg5 and Atg7 in KRAS driven mouse models of pancreatic ductal adenocarcinoma, impedes progression of precursor lesions such as pre-malignant intraepithelial neoplasia (PanINs) to a late stage malignant tumor [30]. Similarly, tissue microarray data of PDAC patient samples reveals significant levels of autophagy related protein expression which, in turn, correlates to poor clinical outcomes [31]. One of the characteristic features of PDAC tumors is upregulation of autophagy under basal conditions and suppression of which averts further PDAC progression, thereby demonstrating the dependence of PDAC on this pathway [29].

PDAC exhibits extensive desmoplasia which is characterized by dense fibrotic stroma, poor vasculature and consequent harsh tumor micro-environmental conditions like hypoxia and nutrient deprivation [32]. Researchers hypothesize that autophagy acts as a salvage system to promote PDAC tumor survival and proliferation during these conditions by contributing to adaption strategies for different metabolic challenges. For example, in PANC-1 and MiaPaCa-2 cells, activation of autophagy through cell survival pathways like MAPK and NF-kB facilitates PDAC cell survival by inhibiting apoptosis [33]. Moreover, findings from two independent studies demonstrate that upregulation of autophagy in Panc-1 and BxPC-3 cells induced either by hypoxia-inducible factor-1α (HIF-1α) or by long noncoding RNA metastasis-associated lung adenocarcinoma transcript 1 (MALAT1) promotes metastatic and proliferative ability of these cells [34, 35]. Autophagic genes and subsequent lysosomal systems are upregulated in PDAC. In physiological conditions of nutrient stress, autophagy onset is under control of MiT/TFE family of transcription factors (MITF, TFE3, TFEB and TFEC), however, during PDAC pathogenesis, nuclear retention of these proteins promotes activation of the autophagasome-lysosome machinery essential for PDA growth regardless of nutrient status [36, 37]. PDAC metabolism can be influenced by autophagy not only by providing metabolic substrates during the time of nutrient starvation but also by maintaining organelle functions (**Figure 1**). A recent study indicates that the key mitochondrial function of oxidative phosphorylation is maintained in an autophagy dependent mechanism [38]. Apart from this, autophagy has also been shown to promote PDAC tumor survival in non-cell-autonomous or cell-extrinsic manner i.e. by supporting the tumor metabolism by modulating other cell types or promoting cancer cachexia. [39]. Autophagy induced secretion of non-essential amino acids via pancreatic stellate cells helps in sustained survival of PDAC in an inhospitable environment [24]. Additionally, it also helps in maintaining pancreatic cancer stem cell activity [40] as well as in regulation of macrophage infiltration thereby facilitating PDAC promotion.

To summarize, autophagy plays a critical role in PDAC metabolism and progression through catabolic degradation of bioenergetic macromolecules and consequently supporting oncogenic events and metabolic adaption-driven pathways required for PDAC survival and proliferation in stressful environmental conditions.

## MACROPINOCYTOSIS: AN OLD MECHANISM WITH A NEW FUNCTION

Cell metabolism adaptability is required for pancreatic cancer to survive in an environment where nutrients and oxygen are scarce. Autophagy is a fundamental pathway to produce nutrients and recycle macromolecules, leading to tumor survival in an exceptionally harsh environment. However, it can't be used by the cells to increase their biomass since autophagy is limited by degradation of intracellular content. Interestingly, PDAC cells also rely on a second lysosomal-dependent pathway of macropinocytosis to fuel their elevated metabolic demand and create a net increase in nutrients availability (**Figure 1**).

One of the main signaling pathways involved in the induction of macropinocytosis is the MAPK pathway. Indeed, PDAC cells carrying a mutant form of KRAS utilize macropinocytosis to incorporate many different nutrients from exogenous origins [41]. For example, tumor cells may use extracellular albumin, since it is the most abundant protein in our blood, or collagen from the extracellular matrix (ECM) to produce glutamine and proline respectively, to support TCA and create the carbon skeleton used for biosynthetic processes [42, 43]. It has been shown that Ras-driven cancers also rely on this mechanism for lipid scavenging, an advantageous process as lipid synthesis is one of the most expensive metabolic pathways for the cells, in terms of oxygen and energy (ATP and NADPH) [41, 44].

The potent ability of the KRAS mutation to stimulate macropinocytosis may be one of the reasons why it is predominantly mutated in pancreatic cancers [6]. However, although KRAS accelerates the rate of the internalization of extracellular fluid into the endosomal compartment, it does not determine lysosomal degradation of the ingested proteins. Indeed, this second step is strictly controlled by the mTORC1 complex. If the essential amino acids are present in the extracellular microenvironment, tumor cells can use transporters to uptake them for their metabolic demand. In these conditions, mTORC1 is activated by the high level of nutrients and localizes to the lysosomes, where it prevents the fusion between endosomes and lysosomes, thus limiting the use of extracellular components to fuel the cell metabolism [45]. Instead, in an essential-amino acids depleted microenvironment, mTORC1 is inhibited by the low level of intracellular nutrients and the cells induce lysosomal catabolism of extracellular proteins in order to survive under this limiting condition [46].

The role of mTORC1 is controversial, it is believed to play a central role in tumor growth, but, in a condition of low nutrients, the cells should be able to suppress the mTORC1 signaling pathway in order to survive. This could be a possible reason for so few mutations being found directly on mTORC1 complex in PDAC tumors, since with a constant activation of this pathway tumor cells are unable to adapt to nutrient deprivation. Finally, this mechanism could also be at the base of the modest successes of mTOR inhibitors, especially in KRAS driven cancer. Indeed, rapamycin analogue treatment could enhance the digestion of extracellular content, supporting cell survival and growth in a nutrient-depleted condition [45, 46].

## EXPLORING NEW THERAPEUTIC PERSPECTIVES

Most of the patients are diagnosed during advanced stages of pancreatic cancer or the early stages of PDAC are usually asymptomatic due to which the overall survival of patients remained unchanged for decades. Only a modest improvement in overall survival was obtained with FOLFIRINOX (folinic acid, 5-fluorouracil, irinotecan, oxaliplatin) or nab-paclitaxel plus gemcitabine chemotherapy [47]. The impairment in drug delivery due to desmoplasia as well as the fast emergence of resistance consequently limit the efficacy of currently available therapies. Therefore, novel strategies of therapeutic interventions are of utmost importance (**Figure 1**).

### Targeting the metabolic rewiring

To meet their demand for energy, cancer cells reorganize their entire metabolism (in particular, glycolysis and the TCA cycle) to augment anabolic reactions linked to cell growth and proliferation. This metabolic reprogramming, including increased glycolysis, TCA cycle and autophagy, represents not only a mechanism for survival but also a putative Achille's heel of pancreatic cancer for therapeutic intervention. Emerging therapeutic strategies include the targeting of key regulators of metabolism, such as pyruvate kinase isoenzyme M2 (PKM2) or lactate transport and autophagy. Inhibition of PKM2, a hub for glycolytic switching, reduced cancer cell growth *in vitro* [48]. The targeting of the lactate production and transport represents another strategy. Although several LDH inhibitors are in preclinical development, their short half-life has limited their efficacy *in vivo*. On the contrary, AZD3965, a MCT-1 lactate transporter inhibitor, is currently being evaluated in a Phase I trial (ClinicalTrials.gov Identifier NCT01791595) for its ability to reduce tumor growth.

Besides anaerobic glycolysis, the metabolism of NAD, a crucial cofactor in redox balance, is critical for pancreatic cancer development [50]. Inhibition of NAD synthase via NAMPT pathway blocks the cell growth and survival of pancreatic tumor both *in vitro* and *in vivo*. The consequent massive reduction in the metabolic activity is accompanied by low levels of NAD, glycolytic flux, lactate production, mitochondrial function and levels of ATP [50]. Abrogating the regeneration of NAD by inhibiting nicotinamide phosphoribosyltransferase disrupts aerobic glycolysis and leads to pancreatic cancer cell growth inhibition *in vitro* and *in vivo*, although no tumor regressions were seen in *in vivo* models.

### Autophagy inhibitors

It has been demonstrated that the genetic and pharmacological inhibition of autophagy delays tumor development in mouse models. One of the most promising drugs appears to be hydroxychloroquine, an autophagy inhibitor approved by the FDA for the treatment of malaria but also commonly used for rheumatic diseases. As a single agent, hydroxychloroquine proved to be inconsistent at autophagy inhibition and demonstrated negligible therapeutic efficacy in patients with pancreatic cancer [49]. However, the combination of hydroxychloroquine with nab-paclitaxel plus gemcitabine chemotherapy is currently undergoing evaluation both in the neoadjuvant setting (ClinicalTrials.gov Identifier NCT01978184) and in advanced-stage disease (ClinicalTrials.gov Identifier NCT01506973; NCT01128296; NCT01494155; NCT01273805).

Given the role of mTOR as a master regulator of autophagy, mTOR inhibitors represented an alternative approach, but, despite the first promising studies, phase II clinical trial failed to demonstrate clinical benefits [50]. Probably, a stronger antitumor effect could result from the inhibition of multiple signaling pathways, as illustrated by the studies on Metformin; an inhibitor of AMPK/mTOR, MAPK, IGF-IR, NFkB pathways. Metformin, a FDA-approved antihyperglycemic drug, is used as first-line therapy for diabetes mellitus type 2. Thanks to the inhibition of *de novo* fatty acid synthesis and the alteration of the PPP and subsequent nucleotides and DNA synthesis, Metformin reduces the risk of PDAC in diabetic patients and has antitumor effects [52]. On the contrary, a randomized phase II trial failed to show a survival benefit for simvastatine, an inhibitor of cholesterol synthesis, in patients treated with gemcitabine [51].

On the other hand, the reduction in cholesterol uptake through Low-Density Lipoprotein Receptor (LDLR) blockade may be an alternative strategy, as it sensitizes cells to chemotherapeutic drugs such as gemcitabine [53].

### Macropinocytosis inhibitors

As previously described, micropinocytosis is adopted by pancreatic cancer in order to sustain tumor propagation. Indeed, this survival mechanism may represent an avenue for novel therapeutics in PDAC.

The currently available pharmacological tools to inhibit macropinocytosis include PI3K-AKT-mTOR inhibitors, actin polymerization inhibitors [54], and sodium/hydrogen exchangers (NHEs) inhibitors [55].

The PI3K inhibitors wortmannin and LY290042 are known to block macropinocytosis and phagocytosis in different cell types [56]. Given the involvement of mTOR in lysosomal degradation and promotion of macropinocytosis, rapamycin analogs could represent a treatment option. Another therapeutic approach includes the disruption of cancer cell metabolic activity through inhibition of the phosphoinositide-3-kinase (PI3K) pathway. It was demonstrated that the class I PI3K inhibitor wortmannin inhibits scission of macropinosomes from the cell surface, reducing pancreatic cancer motility, invasion and metastasis [57].

The actin perturbant cytochalasin D and latrunculins are known to inhibit not only membrane but also several other cellular processes involving actin polymerization, resulting in an aspecific targeting of macropinocytosis [58]. Currently, the selective NHE blockerethyl-isopropyl amiloride (EIPA) and di-methyl amiloride are considered to be the first choices for pharmacological inhibition of macropinocytosis [56]. However, possible endocytosis-unrelated effects of these drugs on ion transport, intracellular pH and cytoskeleton limit their use as pharmacological inhibitors of macropinocytosis.

In addition, a recent study suggests that the concomitant targeting of the glutamine metabolism and macropinocytosis may provide an appropriate therapeutic rationale for KRAS-driven tumors [59]. On the other hand, macropinocytosis can be positively used to enhance the internalization of nanoparticles, carrying small molecules, genetic components or chemotherapeutic drugs [42].

## CONCLUDING REMARKS

The toxicity profile of drugs targeting ubiquitous metabolic enzymes in humans is a major concern. To enable targeting of metabolic treatments and to ensure an adequate toxicity profile, identification of mutations that result in metabolic pathway addiction in cancer cells will be crucial. For example, gemcitabine resistance emergences for the upregulation of genes involved in pyrimidine biosynthesis and HIF overexpression. Inhibition of pyrimidine synthesis and HIF through leflunomide and digoxin respectively, would disable DNA synthesis as well as adaptation to hypoxia, thereby rendering pancreatic cancer sensitive to presumably any therapeutic stress (sensitizing PDAC tumors to gemcitabine) [60]. The inhibition of glutamine metabolism, which regulates redox balance, can sensitize PDAC to oxidative stress, generated by standard chemotherapy used in PDAC patients. These findings can pave the way to new therapeutic strategies for the synergistic application of glutamine inhibitors and therapies able to increase reactive oxygen species (ROS) production, as radiotherapy and chemotherapy [18].

Therapeutic resistance of PDAC to radiotherapy, targeted agents, and chemotherapy means that new therapeutic strategies are urgently needed. Recently, several studies in cancer therapy are exploiting the biochemical differences between cancer cell and normal cell metabolism. The metabolism of pancreatic cancer is profoundly rewired by KRAS mutations, offering new opportunities for selective targeting. Therefore, one avenue would be to target pancreatic cancer nutrient acquisition processes, as a number of studies have linked autophagy and micropinocytosis to tumor survival and progression. While autophagy is a potent and adaptive survival mechanism, cells are ultimately limited by intracellular content. In contrast, macropinocytosis is a way to generate a diverse array of essential building blocks from an exogenous source which can contribute to cellular biomass. Therefore both these processes are crucial for pancreatic cancer cells, providing them with a means to grow in the exceptionally harsh PDAC environment. In addition, considering that these pathways converge at the lysosome, it is also tempting to speculate that lysosomal perturbation could be particularly toxic in PDAC tumors.

Future studies will be needed to gather a more detailed understanding of the molecular events controlling nutrient acquisition in pancreatic cancer. Moreover, studies focused on combination therapies employing autophagy/macropinocytosis inhibitors in conjunction with other metabolic pathway inhibitors could pave the way to improve therapeutic outcomes.

## Abbreviations:

α-KG: α-ketoglutarate
GOT1: Glutamic-oxaloacetic transaminase
mTOR: mammalian target of rapamycin
PDAC: pancreatic ductal adenocarcinoma
PPP: pentose phosphate pathway
TCA: tricarboxylic acid
